# How to Systematically Assess Serious Games Applied to Health Care

**DOI:** 10.2196/games.3825

**Published:** 2014-11-11

**Authors:** Maurits Graafland, Mary Dankbaar, Agali Mert, Joep Lagro, Laura De Wit-Zuurendonk, Stephanie Schuit, Alma Schaafstal, Marlies Schijven

**Affiliations:** ^1^Department of SurgeryAcademic Medical CenterAmsterdamNetherlands; ^2^Desiderius SchoolDepartment of Medical EducationErasmus University Medical CentreRotterdamNetherlands; ^3^National Military Rehabilitation CentreDoornNetherlands; ^4^Department of GeriatricsHaga ZiekenhuisDen HaagNetherlands; ^5^Department of Obstetrics and GynaecologyMáxima Medical CentreVeldhovenNetherlands; ^6^Departments of Emergency Medicine and Internal MedicineErasmus University Medical CentreRotterdamNetherlands; ^7^Department of ICTWindesheim University of Applied SciencesZwolleNetherlands

**Keywords:** consensus, serious game, applied game, telehealth, mobile health, video game

## Abstract

The usefulness and effectiveness of specific serious games in the medical domain is often unclear. This is caused by a lack of supporting evidence on validity of individual games, as well as a lack of publicly available information. Moreover, insufficient understanding of design principles among the individuals and institutions that develop or apply a medical serious game compromises their use. This article provides the first consensus-based framework for the assessment of specific medical serious games. The framework provides 62 items in 5 main themes, aimed at assessing a serious game’s rationale, functionality, validity, and data safety. This will allow caregivers and educators to make balanced choices when applying a serious game for healthcare purposes. Furthermore, the framework provides game manufacturers with standards for the development of new, valid serious games.

## Background

*Serious* or *applied games* are digital games with the purpose to improve individual’s knowledge, skills, or attitudes in the “real” world. Serious games applied to medical or health-related purposes are growing rapidly in numbers and in types of applications. Serious games have been shown to be at least as effective as conventional tests in improving cognitive abilities in the elderly [1] and even more effective than conventional neuropsychological interventions when it comes to improving neuropsychological abilities of alcoholic patients [2]. Serious game-based interventions have been used to support rehabilitation in disabled patients, showing equal effectiveness compared to conventional training programs [3]. Games have been applied to promote healthy behavior in children [4] and educate patients [5,6]. Serious game-based patient education has also been shown to increase the treatment adherence among adolescents with leukemia [7]. A third application for serious games is training medical personnel [8]. Serious games have been shown to add to Advanced Life Support training [9] and improve understanding of geriatrics principles among medical students compared to conventional training methods [10]. Patients [11], students, and professionals [12] generally view game-based interventions as fun and challenging.

Although results for serious games in terms of effectiveness for such purposes are promising, their implementation as “serious” modalities for prevention, treatment, or training in health care is hindered by lack of understanding of the underlying concepts among health care professionals, or even distrust. Before doctors and patients consider using serious games as a useful solution for a health care-related problem, it is important that they understand what problem is being addressed by the game and that a proposed claim on effectiveness is indeed trustworthy. Many clinicians are currently undereducated in judging a serious game’s safety or effectiveness. Information on individual games is often hard to find in disorganized app stores and websites [13]. Studies on serious games’ validity and effectiveness remain scarce [8,14]. The idea of applying a video game in health care may even be resentful to certain clinicians or patients. In addition, threats to data safety fuel distrust towards electronic applications in health care altogether [15]. Such issues menace the practical application of serious games throughout health care, subsequently limiting investments in smart solutions that may actually prove beneficial in the end.

This article discusses the first tool for the systematic assessment of serious games applied to medical use, for educators and clinicians. The information collected and organized accordingly, will aid health care practitioners to understand and appraise the risks and benefits of specific serious games in health care in a uniform manner.

## Assessment Framework

To our knowledge, there is currently no systematic framework for the assessment of serious games in health care described in literature. Therefore, the Dutch Society for Simulation in Healthcare (DSSH) [16] has developed a consensus-based framework, categorizing important items that assess a serious game’s safety and validity. Eight individuals (see Acknowledgements section for details) from six different institutions experienced in designing, applying, or researching serious games for health care-related purposes participated. The reporting standards for *non-*game mobile health apps for medical purposes (mHealth), published by Lewis [17] and Albrecht [18], was used as a basis. This system is applied by the peer-reviewed mHealth app assessment initiative of the *Journal of Medical Internet Research* [19]. Due to inherent differences in the functionality of *games* compared to purely informational *mHealth* applications, this framework required re-evaluation.

The panel reviewed the items from these reporting standards during two meetings. All items in the Albrecht framework [18] were systematically evaluated. For each of the 5 categories, items irrelevant to serious games were removed and if necessary, extra items were added. During the second panel meeting, the framework was re-evaluated and all members approved the final version.

The framework described provides 62 items in 5 main themes (Table 1), aimed at assessing a serious game’s rationale, functionality, validity, and data safety. It specifically does *not* aim to assess its effectiveness in terms of success or user attractiveness. The panel defined serious games (other than a regular medical application) as digital applications instigating a specific behavioral change to its user, in the form of skills, knowledge, or attitudes useful to reality [20]. The framework does therefore *not* apply to (mobile or Web-based) digital health apps with a purely informational purpose, for which the *mHealth* app assessment framework is designed [18].

**Table 1:** Items relevant for the assessment of a serious game used for health care-related purposes.

	Category	Item	Question
Game description	Meta-data	Operating system	Operating systems of the game
		Version	Version
		Web-link	Web-link
		Project type	Commercial, non-commercial, other
		Access	Public / restricted / other
		Adjunct devices	Is an adjunct device needed?
	Development	Funding	How was development funded? Eg, funding agencies, investors
	Sponsoring / Advertising	Advertisement policy	Is the game free of commercial pop-ups?
			If not, what is advertised?
		Sources of income	Are there sources of income within the game?
		Sources of income outside game	What are the sources of income of the owner/distributor?
	Potential conflicts of interest	Affiliations	What affiliations do the publishers have that could influence content or user group?
		Conflicts of interest	What interests do the publishers have that could influence the game’s content or user group?
		Disclosure	Are conflicts of interest disclosed?
Rationale	Purpose	Goal or purpose	What is (are) the purpose(s) of the game?
		Disclosure	Is (are) the purpose(s) disclosed to users?
	Medical device	Medical device	Is the serious game a medical device, or not?
		Class	If yes, which class?
		Approval by legal bodies	If yes, does it comply to the necessary requirements (FDA-approval, CE-mark?).
	User group	Specific user groups	For each user group: disease/condition, or health care profession.
		Description	Please specify gender, age (range), and other relevant descriptive items.
		Limits	Are there age limits, or other limits?
		Disclosure	Is the intended user group disclosed?
	Setting	Patient care	Is the game used in patient care?
		Training courses	Is the game used in training courses or -curricula?
		SCORM compliancy	If used in training courses or curricula, is the serious game SCORM-compliant?
Functionality	Purposes / didactic features	For every purpose of the game:	
		Learning or behavioral goals	What content will the player learn?
		Relation learning and gameplay	How does the learning content relate to the gameplay?
		Instruction	What intervention leads to the learning transition (eg, tutorial, instructions (in-game))
		Assessment (progress) in game	Through which parameters is progress in the game measured?
		Assessment parameters	Which parameters are to designers' opinion indicative for measuring learning effects?
	Content Management	Content Management system	Is the Content Management System restricted to specified persons or institutions?
		User uploaded content	If no, are users allowed to upload their own content?
		Content monitoring	How is uploaded content checked?
		Restrictions and limits of the serious game	Please describe restrictions and limits of the serious game. What content on the learning goals is not covered?
	Potentially undesirable effects	Potentially undesirable effects	What potential undesirable effects could the game have?
		Disclosure	Are such potential undesirable effects disclosed to the user?
		Measures taken	What measures are taken to prevent potential undesirable effects?
Validity	Design process	Medical expert complicity	Were medical experts (content experts) involved in the design process from the start?
		User group complicity	Were representatives from the user group involved in the design process from the start?
		Educationalist complicity	Were educationalists involved in the design process from the start?
	User testing	User testing	Did user testing take place? What were the results, and how were these incorporated in the design?
	Stability	Platform stability	Does the game produce the same results on different platforms?
	Validity (effectiveness)	Face validity	Do educators and trainees view it as a valid way of instruction?
		Content validity	How is its content validated to be complete, correct, and nothing but the intended medical construct?
		Construct validity	Is the game able to measure differences in skills it intends to measure?
		Concurrent validity	How does learning outcome compare to other methods assessing the same medical construct?
		Predictive validity	Does playing the game predict skills improvement in real life?
Data protection	Data protection and privacy	Data processing	How is data collected in the serious game?
		Patient privacy	Are patient-specific data stored in the game?
			If yes, are patient informed consent criteria met according to relevant national standards?
		Data ownership	Who owns and stores the data resulting from play?
		Data storage period	During what period are data stored?
		Data removal	Can the user delete data temporarily and/or permanently?
		Data storage security	Is the data storage secured in conformity with laws of the countries stated above?
		Data transmission security	Is the data transmission secured in conformity with laws of the countries stated above?
		Disclosure	Are all items on “data protection” disclosed to the user?

## Assessing Medical Serious Games

### Game Description

When evaluating a specific serious game, it should be thoroughly described and registered (including information about the manufacturer or owner to whom the game should be attributed and the version). Equally to mobile applications, a special interest is taken into the owner’s policy concerning revenues from sponsoring and advertisements, both during development as well as its use. Sources of revenue and affiliations (eg, pharmaceutical industry) may bias or threaten a serious game’s validity for obvious reasons. These should be fully disclosed to the game’s users. Sources of income within a game can be equally relevant to the costs required for the initial purchase.

### Rationale

This clarifies the game’s purpose outside the game. This external purpose (eg, improving eye-hand coordination in laparoscopic surgery) may differ from the actual goal *in* the game (eg, completing a quest in an underground world [21] or playing a tennis game [22]). This clearly differs from the Albrecht framework, because most mHealth apps have a single obvious purpose (internal goal = external goal). A game’s purpose relates to the intended user group and the setting in which it is used, similar to mHealth apps.

Additionally, serious games might fall within the scope of the medical devices, requiring specific guidelines to be implemented, set by the US Food & Drug Administration (FDA), European Committee (Conformité Européenne*,* CE), or national equivalents. This specifically applies to games with a distinct diagnostic or therapeutic purpose. Moreover, integration of serious games into electronic learning environments may demand certain technical requirements. The industry has set standards to improve the interoperability of e-learning content (the Sharable Content Object Reference Model; SCORM)[23]. Its implementation will improve the integration of educative serious games in learning management software.

### Functionality

Functionality of a serious game clearly differs from that of an mHealth app. These usually contain “dry” content (eg, medical information) or an obvious functionality (eg, communicating or registering information), whereas a game requires the user to operate or interact with the content, with the ultimate goal to change ones behavior in real life (ie, learning). To understanding this process, information is required on the game’s content, how the instruction is delivered, how performance is assessed and how these aspects are integrated in the gameplay [24,25].

Consequently, it is important to register information on the game’s content management. For instance, users may be able to add content themselves, making content validation an important issue. This directly influences the game’s content’s validity.

Finally, undesired results or negative transfer of learning could occur in the interaction with a serious game, which is not the same concept as “gaming the game” (ie, cheating), an effect that may very well enhance learning [24]. If validation research is not present, at least a logical connection between gameplay and behavioral or learning goals should be present and disclosed by the developer.

### Validity

Validity determines whether an instructional instrument (such as a serious game) adequately resembles the construct it aims to educate or measure*.* More formally, “the degree to which evidence and theory supports the interpretations of [game] scores entailed by the proposed use of [the game]” [26]. The American Psychological Association has set a series of standards to measure validity [26]. Whereas many validity types have been described, validity research in medical education usually contains several consequential phases [27,28]. First, experts should scrutinize the game’s content to determine its legitimacy (*content* validity). Second, experts and novices judge the instrument’s apparent similarity to the construct it attempts to represent (*face* validity). *Construct* validity reflects the ability of the instrument to actually measure what it intends to measure (ie, the difference in performance between groups of users with different levels of experience in reality). *Concurrent* validity reflects the correlation between performance on the serious game and their performance on an instrument believed to measure the same construct (eg, a simulator or course). The ultimate goal is to prove a game’s *predictive* validity: does performance in the game lead to better outcomes in reality? Most validation research currently published in the medical domain uses these concepts [29]. For individual cases, relevance of specific validity types may differ. When considering mHealth apps in general, content validity may be the sole source of validity.

Validity research is frequently a long and costly enterprise. Many newly developed serious games have therefore not yet undergone validity research [8]. The framework therefore determines a number of steps to pre-assess a serious game’s potential as a valid instrument, with regard to its design and initial testing phases. This encompasses the involvement of user groups, content experts, or educationalists in the design (if relevant to the game’s purpose). Next, if a game has undergone user testing and stability testing, the game is more likely to have higher face- and content validity.

### Data Protection

Threats to user privacy are imminent in electronic and mobile health apps, especially when patient-specific data are measured or entered in the game [15]. This considers data “at rest” on devices or servers, as well as data “in transit”. It must be clear whether data is collected by the game, who owns the data and whether users can request to remove their data. Storage and analysis of personal data should be disclosed to users and must be in conformity with the laws applied in countries the serious game is distributed in. Special care must be taken if patient information is collected. These items are in general conformity with the requirements for mHealth apps described in Albrecht’s framework [18].

## Discussion

When using serious games in health care, end users (clinicians, patients, or educators) must decide whether games are safe and effective enough to be used for their intended purposes. In order to do so, they need consistent, transparent, and reliable assessments. Are applied games really stating their claim in this field? In the framework described in this article, both developers and end users are supported in assessing relevance, validity, and data safety of an applied game. In order to become a “qualified game”, developers should disclose comprehensive information on their products and claims. They must provide transparency to meet the standards. The *Journal of Medical Internet Research* and the Dutch Society for Simulation in Healthcare [16] have launched an international peer-reviewing initiative for serious games in health care.

The safe application of technology-enhanced solution remains the responsibility of the health care provider. Choosing if a serious game answers to the user’s needs, can be based on information concerning 5 main areas described in this article. The majority of the items cannot be assessed using objective parameters. For instance, claiming a specific serious game’s predictive validity should be supported by solid evidence. A comprehensive evaluation by a panel of experts in the form of a quality label could form a more practical solution.

Guidelines have been recently published reporting standards to support clinicians and patients in distinguishing high quality mhealth apps [17-19] and medical websites [30]. These standards form the basis for the framework described in this article. These standards have two important shortcomings when it comes to games. First, explicit information on a serious game’s content and didactic features is required, as the external purpose of a serious game is frequently less obvious to the user than in the case of mHealth apps. Second, serious games require additional validation steps (eg, construct and predictive validity), compared to non-interactive information platforms. Gameplay is dynamic and learning goals in gameplay are often not disclosed to the user. In fact, the user learns by playing the game, whereas discovery in itself may be part of the gameplay. Disclosing learning goals would thus be counterproductive.

There are several limitations to the framework described in this study. It considers validity of the serious game’s content and its didactic functionality. Validity does not predict a game’s success nor its attractiveness to the user, which also depend on its entertainment capability and distribution method [31]. It does not wish to objectify which game is most fun, but merely which game is most valid. A second consideration is that in the scientific field of validity research in medicine, validity concepts other than the one used in this framework have been proposed [32]. The “classical” validity concepts (content-, face-, construct-, concurrent-, and predictive validity) have been most frequently used in validity research in medicine and therefore the most logical to encompass in the framework presented in this article [27,28].

In summary, this consensus-based tool provides the end users the support required when assessing the effectiveness and relevance of serious games in health care. An FDA-approval or CE-mark is simply insufficient for this purpose. In order to prevent wrongful application and data theft of unsuspecting patients or medical students, this information on medical serious games should become publically available to all end users. This will aid the prescription of safe and effective games to patients and the implementation of games into educational programs.
